# Mobile Delivery of Treatment for Alcohol Use Disorders: A Review of the Literature

**DOI:** 10.35946/arcr.v36.1.11

**Published:** 2014

**Authors:** Andrew Quanbeck, Ming-Yuan Chih, Andrew Isham, Roberta Johnson, David Gustafson

**Affiliations:** Andrew Quanbeck, Ph.D., is a research scientist; Andrew Isham, M.S., is a researcher; Roberta Johnson, M.A., M.Ed., is a senior editor; and David Gustafson, Ph.D, is a research professor and Director, all at the Center for Health Enhancement Systems Studies, University of Wisconsin–Madison, Madison, Wisconsin. Ming-Yuan Chih, Ph.D., M.H.A., is an assistant professor in the Department of Clinical Sciences at the College of Health Sciences at the University of Kentucky, Lexington, Kentucky.

**Keywords:** Alcohol consumption, alcohol use disorders, intervention, treatment, continuing care, electronic health technology, mobile health technology, mobile phone, smart-phone, Internet, telecommunication, literature review

## Abstract

Several systems for treating alcohol-use disorders (AUDs) exist that operate on mobile phones. These systems are categorized into four groups: text-messaging monitoring and reminder systems, text-messaging intervention systems, comprehensive recovery management systems, and game-based systems. Text-messaging monitoring and reminder systems deliver reminders and prompt reporting of alcohol consumption, enabling continuous monitoring of alcohol use. Text-messaging intervention systems additionally deliver text messages designed to promote abstinence and recovery. Comprehensive recovery management systems use the capabilities of smart-phones to provide a variety of tools and services that can be tailored to individuals, including in-the-moment assessments and access to peer discussion groups. Game-based systems engage the user using video games. Although many commercial applications for treatment of AUDs exist, few (if any) have empirical evidence of effectiveness. The available evidence suggests that although texting-based applications may have beneficial effects, they are probably insufficient as interventions for AUDs. Comprehensive recovery management systems have the strongest theoretical base and have yielded the strongest and longest-lasting effects, but challenges remain, including cost, understanding which features account for effects, and keeping up with technological advances.

The advent of mobile-phone technology has been one of the most influential technological advances in world history. In 2014, the International Telecommunications Union (ITU) estimated that the number of mobile-phone subscriptions worldwide (including both personal and business subscriptions) would reach about 7.0 billion at the end of 2014 and thus approach the number of people on Earth (corresponding to a global penetration rate of 96 percent) (ITU 2014).

Furthermore, Google’s “Our Mobile Planet”—a marketing survey commissioned by Google to assess worldwide use of mobile technology—indicated that the use of smartphones (i.e., mobile phones with computer-like capabilities) has increased significantly in recent years (Google, Inc. 2013). According to the survey, more than 50 percent of the population in most developed countries used smartphones in 2013, and rates of smartphone ownership have been increasing steadily year after year. In addition to their many other uses, mobile phones offer an opportunity to monitor various behaviors of their users, such as alcohol consumption, and to deliver interventions to users in near–real time and in the individual’s natural environment. Several review and commentary articles about the use of mobile health (mHealth) and Internet technology in health care, and specifically in the treatment of alcohol use disorders (AUDs), have been published in recent years (Bewick et al. 2008; Carey et al. 2009; Gustafson et al. 2011, 2014; Hester and Miller 2006; Kypri et al. 2005; Savic et al. 2013).

A plethora of research supports the conceptualization of addiction as a chronic, relapsing disease (Bradizza et al. 2006; Brownell et al. 1986; Dennis et al. 2003; Donovan 1996; Lowman et al. 1996; McKay and Weiss 2001; McLellan 2002; Mueller et al. 2007; Witkiewitz and Marlatt 2004). As with other chronic diseases, patient self-management and continuing care are fundamental to effective treatment (Wagner et al. 1996). Although research supports the effectiveness of continuing care in addiction treatment (McKay 2005; McLellan et al. 2005; Simpson 2004), the field historically has offered little ongoing support to patients, whether during treatment when the patient is outside of the clinic walls or after the patient has completed treatment (McLellan et al. 2000; White et al. 2002). Mobile technology may make it possible to provide both self-management help and continuing care more widely.

This article explores the following questions about mobile applications intended for patients dealing with AUDs:

- What mHealth applications to treat AUDs exist that have been evaluated in the peer-reviewed literature and how can they be categorized?
- What are common features of these applications?
- How effective are currently commercially available mHealth applications for AUDs?
- What are the characteristics, benefits, and limitations of mHealth applications for AUDs?
- What is the theoretical grounding underlying these applications?
- What are the challenges and opportunities facing mHealth approaches for AUDs?

By design, this discussion is limited to systems that (1) use mobile technology (i.e., do not rely solely on Web-based approaches); (2) focus on AUDs and not on tobacco or other drugs; and (3) have been evaluated in the peer-reviewed literature.

## Identifying mHealth Applications to Treat AUDs

To identify mobile applications for AUDs, the authors of this article searched electronic databases of the peer-reviewed research literature.^1^ To further identify relevant studies, they also examined the reference lists of the initially retrieved studies. Because the field is changing so rapidly and the discussion should focus on the current state, the initial search only included studies and reports published since 2009. A subsequent expansion of the search to studies published in earlier years (i.e., between 2002 and 2009) yielded no additional results. Based on the abstracts of the identified studies, a final list of studies was created for in-depth analysis. Despite this broad search approach, however, it is possible that some mHealth systems were missed, especially more recent ones derived from currently funded research endeavors that have not yet published their results or descriptions of their systems.

The initial literature search yielded a total of 486 articles, the vast majority of which upon closer inspection were not germane to the issue of mobile treatment for AUDs. Other articles were excluded because they were reviews rather than original studies, did not report results of specific applications, or had not been published in peer-reviewed journals. (More detailed information on the selection process of the articles chosen for further analysis is available from the authors of this article.) Ultimately, the following description and review of mHealth applications for AUDs was based on a set of 20 published studies that describe 14 unique mobile systems, including their originators, names (if applicable), key features, how they were tested, theories on which they are based, target populations, and results (see table). If possible, special attention was paid to the theories on which the systems were based because theory-based development of mHealth interventions may yield more durable and relevant results (Baker et al. 2014).

Of the 14 identified systems, 11 delivered interventions that relied primarily on text-messaging technology. Two systems were designed for smartphones and offered a more comprehensive approach. One system had users play games on mobile devices. For the following discussion, the 14 systems were divided (somewhat arbitrarily) into four categories:^2^

- Text-messaging monitoring and reminder systems that primarily use the mobile phones’ text-messaging capabilities to monitor alcohol use or remind the user to report their alcohol consumption;
- Text-messaging intervention systems that, in addition to monitoring alcohol use, deliver text messages intended to promote abstinence and recovery;
- Comprehensive recovery management systems that use the internal sensors (e.g., monitoring of GPS coordinates) and other computer-like capabilities of modern smartphones to deliver multifaceted messages and interventions; and
- Game-based systems that attempt to engage the user through game playing.

## Text-Messaging Monitoring and Reminder Systems

Several mobile systems have been studied that rely upon texting to deliver reminders and to prompt reporting of alcohol consumption. Keeping track of alcohol use and associated symptoms via text messages or Web-based formats seems to be widely accepted among patients, with high response rates. Thus, an application that used text messages to collect data from patients about their drinking had a response rate of 84.4 percent (Kuntsche and Robert 2009), similar to the response rate of 88 percent reported for an application that used texting as a means of delivering a brief alcohol intervention (Irvine et al. 2012).

Self-assessments using mHealth approaches can provide patients and their counselors with a way to continually monitor patient recovery. One such text-based assessment system is called ICAT; it can be used to collect patient self-assessment data on drinking (Kuntsche and Labhart 2012) as well as their motives for drinking (Kuntsche and Labhart 2013). Bernhardt and colleagues (2005, 2007, 2009) developed another system that uses automatic texting and phone messages as reminders to encourage college students to submit a daily electronic alcohol use assessment via mobile phones; their research focuses on the validation of texting as an electronic assessment method, not on possible interventions. Such mHealth monitoring and assessment tools can be used for various practical applications. For example, Tiplady and colleagues (2009) have used texting in alcohol research to send reminders and assessments to study participants that were related to performing cognitive tasks. Moore and colleagues (2013) used texting primarily as a surveillance tool, although in this case the mHealth application also offered a limited intervention by providing users with feedback on how much money they likely were spending on alcohol given their self-reported consumption.

In general, reminder systems that focus primarily on monitoring consumption do not seem to be effective in reducing alcohol use. Although these systems are not specifically intended to reduce consumption, it could be argued that the process of monitoring alcohol use itself could lead to a reduction in drinking. However, this issue is not likely to receive much more research attention, because basic texting systems are increasingly being supplanted by approaches that focus on the more sophisticated capabilities of smart-phone-based applications. Any text-based reminder systems that are being implemented, however, should also include a confirmation step to increase efficacy. Thus, reminders alone are unlikely to be highly effective unless the recipient of the message confirms that the recommended actions have taken place.

## Text-Messaging Intervention Systems

Several mHealth systems exist that provide targeted interventions to their users. Agyapong and colleagues (2012) developed a message-based intervention that twice a day delivered personalized supportive text messages to patients with AUDs and comorbid depression. This intervention, which was provided for 3 months, led to reduced depression and better cumulative abstinence at 3 months. However, these effects were not observed at the 6-month followup, 3 months after the end of the intervention (Agyapong et al. 2013).

Irvine and colleagues (2012) evaluated a brief alcohol intervention delivered via text messages but focused on the users’ engagement with the intervention (e.g., they assessed whether and how participants used the intervention-related text messages) rather than on the intervention per se. The analysis demonstrated that text messaging can be used not only to deliver an intervention but also to evaluate specific aspects of the treatment process, such as participant engagement with and reaction to intervention components when treatment is delivered via a mobile delivery platform.

Another mHealth intervention, the AGATE system (Stoner and Hendershot 2012), uses tailored texting frequency to promote adherence to pharmacotherapy for addiction treatment by reminding participants to take their medication and confirming that medications are taken. The frequency of these reminders can be adjusted based on adherence rates. For example, if a patient achieves a predetermined goal of medication adherence (e.g., 90 percent of scheduled doses over 2 weeks), the frequency of the texted reminders can be reduced. Although results of a clinical trial testing the effectiveness of this system have not yet been published, the intervention’s design should help clarify whether both reminders and confirmation of reminder receipt are important for promoting medication adherence.

Another innovative mobile-phone-based intervention application involved a contingency management component to reinforce alcohol abstinence (Alessi and Petry 2013). In this study, texting was used to remind patients to take a breath alcohol concentration (BrAC) test, which the patients video recorded using their mobile phones. The video and BrAC data were electronically submitted in real time to the study organizers. The contingency-management portion of the intervention involved a reward that also was delivered via text messaging if the BrAC results were submitted on time and negative. The study found that those patients who received the reminder messages had a higher percentage of negative breathalyzer tests than those who did not. The study demonstrated the feasibility of using mobile phones to support a contingency-management intervention, based on real-time behavioral assessment in the natural environment and timely provision of reinforcement.

Several studies have examined the effects of tailored text messaging on alcohol use. The feasibility of delivering a text-based goal-setting and feedback system to reduce heavy drinking was demonstrated in a study of young adults presenting to the emergency department (Suffoletto et al. 2012). This trial showed promising results in reducing heavy-drinking days and drinks per drinking day, with a larger trial indicating small reductions in self-reported binge drinking and the number of drinks consumed per drinking day over 12-week intervention (Suffoletto et al. 2014). Weitzel and colleagues (2007) were the first to determine the efficacy of tailored text messaging. Their pilot trial found that drinkers who received tailored messages after filling out daily surveys about their drinking behavior had fewer drinks and were less likely to expect getting into trouble because of their drinking than were drinkers who filled out the same surveys but received no feedback messages. A more recent intervention using tailored text messages was based on motivational interviewing principles along with social-networking counseling (Mason et al. 2014). The investigators found that their tailored message intervention may increase the readiness of drinkers to change drinking behavior.

Overall, the text-based intervention systems described here have shown mixed results regarding effectiveness. Although some studies reported positive results (e.g., Suffoletto et al. 2012), most of these studies have been of short duration and only involved relatively small numbers of participants. Conversely, the arguably best designed study by Agyapong and colleagues (2012, 2013) showed little long-term effect.

## Comprehensive Recovery Management Systems

The literature search identified two comprehensive mHealth recovery systems, LBMI-A (Dulin et al. 2013) and A-CHESS (Gustafson et al. 2014). Both systems operate on smart-phones and comprise a variety of tools and services that utilize the capabilities characteristic of such mobile devices, including broadband Internet connection, interactive multimedia applications, text messages, GPS location awareness, and social networking, which have been shown to improve recovery outcomes (Gustafson et al. 2014). For example, both systems include user self-assessment and feedback, a GPS-based tool to warn users when they approach high-risk locations (e.g., a bar they used to frequent), various strategies for coping with cravings, lists of healthy activities, and methods of communicating with supportive others. Many of these resources can be tailored to the specific needs and preferences of the individual user. However, the two systems were developed based on different assumptions about the relationship between mHealth technology and the addiction treatment system.

### LBMI-A

Dulin and colleagues (2013) were influenced by two findings from previous research when developing the LBMI-A. First, the vast majority of people with diagnosable alcohol dependence do not receive treatment, in large part because of the stigma associated with attending traditional alcohol treatment and other barriers that keep individuals from accessing services (Cohen et al. 2007; Grant et al. 2007). Second, even individuals who are not willing to enter formal treatment may be receptive to using interactive Web sites related to alcohol reduction, and using the technology can increase their motivation to change (Lieberman and Huang 2008). Based on these observations, Dulin and colleagues (2013) created a self-administered, portable alternative to traditional treatment. The LBMI-A design was oriented toward motivating a change in drinking through enhanced awareness of drinking and drinking-related problems and providing intervention options for the user to choose from (Dulin et al. 2013). The system includes modules designed to enhance awareness of a drinking problem through assessment and feedback as well as daily interviews about alcohol use. In the daily interviews, users report triggers they experienced and if they drank in response to them. These responses are summarized in a weekly feedback report. Users also receive suggestions and tools for managing their triggers, as well as other issues that could lead to resumed drinking, such as cravings and psychological distress. Additionally, the system focuses on developing social support through an intervention module that encourages users to identify individuals in their social network who they can turn to for support. If users choose, they can share their initial feedback reports with their support team (which could include a health-care provider). A pilot study that included 28 individuals who met DSM–5 criteria for an AUD, were drinking heavily, and were not engaged in another form of treatment produced encouraging early results regarding the system’s effectiveness (Dulin et al. 2014). Thus, participants who utilized the LBMI-A system reduced the number of days spent drinking hazardously by approximately 60 percent over the course of 6 weeks, and the number of drinks per day dropped from a mean of 5.6 at baseline to 2.9 while using the system, producing a large effect size (Cohen’s d =1.1). This study also contained a qualitative component in which participants were queried about aspects of the system they found helpful and not helpful. The results of this component have driven the creation of a new app called Step Away that currently is running on an iPhone platform (Dulin et al. 2014). LBMI-A is currently being tested in a clinical trial.

### A-CHESS

In contrast to LBMI-A, A-CHESS is designed to be integrated into the traditional treatment system. The A-CHESS design process was informed by a series of patient/user assessments, the results of which were organized around Marlatt’s relapse prevention model (Brownell et al. 1986; Lowman et al. 1996; Witkiewitz and Marlatt 2004). Training in its use begins before the patient is discharged from residential treatment, so that the patient is familiar with the program’s various features and can use relevant content once back in the community. The patient’s counselor sets up the device so that the information and settings are tailored to the patient and his or her specific situation and interests. For example, set-up information includes the patient’s therapeutic goals and care plan, his or her triggers and high-risk situations for drinking, healthy activities the patient is interested in, or benefits the patient expects from sobriety. Services provided by the A-CHESS system include contacts for emergency (i.e., when the patient is at immediate risk of relapse) and nonemergency situations (e.g., weekly check-ins), triage and feedback through various resources (e.g., coping skills, diversionary activities), social support (e.g., discussion groups, contacts with experts), and information services. Through these services, A-CHESS can help patients meet the challenges they often face in life, such as loneliness and isolation, transportation problems, difficulties managing the treatment regimen, and lack of informal support. A-CHESS also addresses such issues as craving and insufficient coping skills in high-risk situations. Additionally, A-CHESS includes a service that—with patient permission—reports patient responses to a weekly Brief Alcohol Monitor that warns clinic staff of imminent relapse and signals a need for clinical intervention (Chih et al. 2014). This clinician-reporting function was included because previous work had shown that a clinician report could facilitate earlier interventions (Dubenske et al. 2008) and that patients whose caregivers could communicate patient symptoms to clinicians had less symptom distress than patients without access to a clinician report (Dubenske et al. 2010). A-CHESS currently is being extended to include a cognitive behavioral therapy–based treatment component (Marsch et al. 2014) for implementation in primary care settings (Quanbeck et al. 2014).

The efficacy of A-CHESS was evaluated in a randomized clinical trial comparing patients using A-CHESS with a control group receiving treatment as usual. The trial found that patients assigned to A-CHESS had 57 percent fewer heavy drinking days compared with the control group (Gustafson et al. 2014). Analyses of the possible mechanisms that may underlie A-CHESS effects indicated that the mobile intervention, delivered in the natural environment as part of continuing care, seems to reduce risky drinking by enhancing the patient’s perceived competence (Gustafson et al. 2014), a construct similar to self-efficacy.

Other analyses of A-CHESS have explored how the data generated by such a mobile intervention (e.g., the data obtained from the weekly “check-in” function that tracks the recovery process) can be used to predict relapse risk and tailor the intervention accordingly to the needs of the patient (Chih et al. 2014). Using more than 2,900 weekly responses from 152 patients, the model was shown to have good ability to predict relapse. Although challenges still exist in analyzing large, complex, time-intensive datasets such as the ones generated by A-CHESS, the predictive model is a step toward “just-in-time” and adaptive interventions that provide support when and where patients need it most.

## Game-Based Systems

Modern mobile devices such as smartphones have various capabilities not found in traditional mobile phones, including the capacity to provide multimedia applications, such as streaming videos and gaming. In a recent randomized controlled trial, Gamito and colleagues (2014) compared a mobile-delivered, gaming-based, neuropsychological intervention plus treatment as usual with treatment as usual only in a sample of alcohol-dependent patients. The results indicated that the addition of cognitive games delivered via a mobile device (i.e., an Android tablet) to treatment as usual helped to improve certain cognitive functions, specifically those associated with frontal lobe–related impairment. Although the intervention effects were somewhat limited, the results suggest that mobile delivery of a game-based neuropsychological intervention, which can help engage patients and provide intervention “on demand,” may help improve certain aspects of cognitive functioning among alcohol-dependent patients. However, current development in this area is still in its infancy (Gamito et al. 2014).

Other mobile applications and capacities, such as sensors, have not been widely studied. Sensors on wireless-connected mobile devices generally hold the potential to enhance continuous monitoring and instant support to addiction patients. However, further development and research are needed in order to provide evidence for clinical applications.

## Commercial Applications

In addition to the applications discussed here, a plethora of other commercial applications are available for smartphone users via Apple’s app store and the Google Play store that have not been evaluated in the peer-reviewed literature. Two recent reviews of alcohol treatment applications found in these online marketplaces have summarized the functions and features as well as the underlying evidence base of these commercial systems. Cohn and colleagues (2011) reviewed 222 apps available in the Apple app store that intervene on alcohol use. The review focused on codifying the principles and evidence base underlying the applications. Subsequently, Savic and colleagues (2013) evaluated 87 apps available in the Google Play store that were aimed at recovery from both AUDs and addiction to other drugs, focusing on the applications’ features and functions. Taken together, the two analyses allowed for the following conclusions:

- The most common features of apps were information on recovery, motivational content, social support tools, and tools for monitoring alcohol use.
- Few of the recovery apps found in both market places were reported to have been created by clinical experts.
- Apps that claim to function as interventions provided little or no empirical evidence of effectiveness.
- Quality control seems to be a concern and an important barrier to use; in the review section of the Google Play store, the most common criticisms concerned technical glitches (22.1 percent) and improvements needed (21.0 percent).

Although some apps include features that reflect empirically based treatment (including motivational enhancement, coping/self-control training, social skills training, and/or cognitive therapy components), very few report that they have been designed according to evidence-based practices. However, citing evidence-based practice may not be an effective marketing strategy in such a direct-to-consumer model. In these marketplaces, users may be more likely to purchase an app based on factors such as the number of downloads and user ratings.

In sum, the evidence base used to develop most commercial systems, as well as empirical tests of their efficacy, are insufficient, despite the popularity and availability of these systems. This commercialization of health products or applications with unproven efficacy is of concern from a public health perspective. To address this concern, researchers might consider conducting comparative studies of some of these applications, particularly those that seem to be more promising based on their underlying theoretical grounding. As mHealth technologies are evolving, reviews of the available commercial systems and their efficacy, such as those conducted by Savic and colleagues (2013) and Cohn and colleagues (2011), should be updated regularly.

## Characteristics, Advantages, and Limitations of mHealth Systems

### Technology, Complexity, and Integration

The mHealth systems described in this article cover a broad spectrum of complexity, ranging from relatively simple text-based monitoring and reminder systems to comprehensive recovery management support systems. In general, the less complex text-based systems were designed with minimal theoretical grounding. With the addition of more diverse intervention functions to create more comprehensive systems, however, communication, behavioral, and social support theories increasingly were used to inform the design of these functions. In general, both simple and complex systems have their advantages and disadvantages.

Systems that rely primarily on texting for monitoring and intervention have the advantage of being inexpensive and widely available, given the nearly universal penetration of basic mobile phones. Moreover, they are easy to operate for both senders and receivers of text messages. These characteristics make it relatively easy to incorporate text-based approaches into existing treatment. For example, text-based reminders are relatively common in addiction treatment and in daily life. Treatment providers can easily avail themselves of free, Web-based systems that automatically generate text-messaging reminders for appointments, medications, or other tasks a provider deems important for a patient to self-manage (see www.ohdontforget.com for an example of such texting software). An example of a text-based reminder system used in a health-care setting (although not in the realm of mobile treatment for AUDs) is a system called text4baby (see www.text4baby.org) that was developed to promote the health of pregnant women and their unborn children. The system has been widely used and evaluated. Studies of the system have suggested that text messages need to be timely and relevant to be valuable to users, a requirement that may lend itself more readily to relatively predictable health episodes, like pregnancy, than to chronic and relapsing conditions such as AUDs.

The main disadvantage of texting-based systems to date is that evidence of their effectiveness is rather limited. The studies reviewed for the preparation of this article showed only limited effectiveness of the text-based interventions for AUDs and only involved relatively small trials of short duration. For instance, the studies by Agyapong and colleagues (2012, 2013) evaluated a 3-month intervention and 3-month followup among 54 patients. Statistically significant effects on depression scores were observed at 3 months, as well as a trend toward increased abstinence, but these effects had dissipated by the 6-month mark (after the intervention was removed). Beyond alcohol treatment, recent evidence has suggested that text4baby has had little success in changing health behaviors (Evans et al. 2014). The available evidence thus suggests that texting-based applications alone probably are insufficient as interventions for AUDs, although it is possible that longer interventions could produce longer-lasting effects. Nevertheless, text-messaging could serve important functions as a component of more comprehensive systems.

Of the various mobile systems tested thus far, the comprehensive A-CHESS system has had the strongest and longest lasting effects, including a reduction in heavy-drinking days of 57 percent, compared with a control group, over an 8-month intervention and 4-month follow-up period (Gustafson et al. 2014). Compared with simple text-messaging interventions, more complex applications that combine various comprehensive training and support tools may produce more substantial and lasting effects. One potential explanation for this greater effectiveness is that a comprehensive application can provide more modes of treatment and tools, such as appropriate contact information for people who can support the user in different risk situations, GPS-data– based warnings of potential high-risk locations, suggestions for alternative activities, or different coping tools. This wide variety of options and tools allows the system to better address the individual user’s preferences in terms of coping styles and interests, leading to better learning and longer-lasting recovery.

However, the enhanced features and effectiveness of comprehensive systems also are associated with increased costs. Although smartphone use is proliferating, owning and operating a smartphone still is considerably more expensive compared with standard cellular phones. Moreover, designing these comprehensive systems requires skilled computer programmers, who must be retained to maintain and improve the system over time, also contributing to the systems’ overall costs. To date, no studies have compared the costs and effects of texting interventions vs. comprehensive mHealth systems.

### Theoretical Grounding

The level of theoretical support for the various applications analyzed in this literature review varied greatly. Particularly for those applications that could be characterized as text-message monitoring and reminder systems, the reviewed studies provided minimal theoretical grounding. The studies that assessed text messages as an intervention approach (rather than just for reminders and monitoring of alcohol use) were more likely to be based on a theoretical framework. For example, several of these studies designed text messages based on theories in communication and behavioral sciences, such as the social-cognition model and motivational-interviewing methods, to improve participants’ mood and offer support for abstinence or reducing alcohol use. Studies that collected feedback from patients (e.g., via texting, Web forms, or e-mail) often employed empirically validated methods, such as contingency management, medication adherence, or guidelines for brief intervention recommended by the National Institute on Alcohol Abuse and Alcoholism, to generate customized messages based on patient responses. Both LBMI-A and A-CHESS were designed as comprehensive recovery-management support systems and are supported by well-established theories about addiction recovery. Thus, in addition to theories based in communication and behavioral sciences, both of these comprehensive systems incorporate social–support-based theories, such as community reinforcement (Dulin et al. 2013) and self-determination theory (Ryan and Deci 2000).

One should note, however, that the concept of theory-based developments may be a double-edged sword. On one hand, established theories can provide a structure that can guide the development mHealth systems. For example, during the development of the A-CHESS system, the developers based their approach on self-determination theory (Ryan and Deci 2000), which states that quality of life is determined by three domains—social relatedness, coping competence, and intrinsic motivation. An understanding of the concepts of this theory can provide a structure for the design of such a system and can suggest ways of achieving goals in each domain. Thus, acknowledgement of the theory might suggest ways in which technology could help develop coping competence so that the user gains the confidence that he or she can cope with stressors that arise. On the other hand, overly strict adherence to theory can be restrictive and may lead to a disregard of the real-life needs, experiences, and struggles of both the patients and the treatment providers involved in their care. In some cases, the involvement of experts from outside disciplines with innovative approaches can add new dimensions to such programs that address the actual needs of the patients and their care providers. Thus, to design effective mHealth applications, it is necessary to strike a balance between adhering to theory and incorporating innovative outreach approaches that can help ensure that the system is appealing to patients and treatment providers in the real world.

## Challenges and Opportunities

Developing and executing mHealth applications, whether they are research driven or commercial, are extraordinarily challenging processes. Users increasingly expect applications to be intuitively designed (so that they require little or no instruction), to provide feedback confirming data transfers, to provide notifications about new actions to take, and so on. Applications that fall short of these expectations are unlikely to be used regularly and, consequently, to be effective. Building a well-designed, adaptable, seamless application requires extensive technical resources, including hardware, software, and programming support. As a result, it is difficult to develop and maintain effective, yet inexpensive, mHealth systems for small populations or short-term goals. Additionally, cost is a concern not only in terms of development but also in terms of availability to patients. Although cellular phones have become commonplace, smartphones that allow the most comprehensive applications may be less available to low-income patients.

Another challenge is that although many features are available in mHealth applications, it is not known which of these are responsible for any observed effects (i.e., are the “active ingredients”) or which features might be most important for different types of patients. Research will need to address these questions.

Finally, technological advances proceed so swiftly that research can hardly keep pace; by the time results from a randomized clinical trial are available and published, the application studied may already be outdated (and, possibly, its results as well) (Baker et al. 2014). Nevertheless, this rapid progress also offers opportunities. For example, continuously evolving technology will make it possible to include new tools and services in mHealth applications, such as wirelessly connecting an application to BrAC testing (Alessi and Petry 2013). Other potential features and applications may include the use of data from mHealth systems to create models that predict relapse (Chih et al. 2014) and initiate measures to prevent its occurrence; multimedia delivery of interventions (Gustafson et al. 2014); and tailored delivery of intervention components to make the applications optimally effective (Gustafson et al. 2014; Mason et al. 2014; Suffoletto et al. 2012; Weitzel et al. 2007). The gold standard of scientific evidence—the randomized trial—may be an unrealistically high bar in this fast-changing field that already is saturated with commercial applications that lack evidence (Baker et al. 2014; Cohn et al. 2011). Instead, researchers could use statistically efficient designs (such as fractional-factorial and quasi-experimental designs) as well as surrogate endpoints to evaluate interventions and delivery systems already in use. Thus, the pace of technological advances offers both a challenge to researchers and great promise for the development of new and effective mHealth approaches.

## Figures and Tables

**Table:** Summary of Peer-Reviewed Mobile Application Systems to Treat Alcohol Use Disorders

Originator & Lead-Author Affiliation	Name	Features	Design	Theoretical/Empirical Basis	Target Population	Results	Reference(s)
**Text-Messaging Monitoring and Reminder Systems**							
Kuntsche and colleagues, Switzerland Research Institute on Addiction, Lausanne, Switzerland	Internet-based, cell phone– optimized assessment technique (ICAT)	Frequent text messages with hyperlinks to questionnaires on weekend alcohol consumption	Survey (*n* = 183)	None noted	College students	High retention rate; alcohol consumption similar to Internet-based assessment. Data collected via ICAT helped clarify the relationship between motive at pretest and alcohol consumption.	Kuntsche and Robert 2009; Kuntsche and Labhart 2012, 2013
Bernhardt and colleagues, Centers for Disease Control and Prevention, Atlanta, Georgia	Handheldassisted network diary (HAND)	Self-reported alcohol consumption using a daily diary administered via mobile phone	Randomized controlled trial (RCT) (*n* = 168) Intervention: HAND Control: paper-and-pencil daily social diary	None noted	College students	HAND assessment reported similar level of total drinks, drinking days, and drinks per drinking days as paper-based daily social diary over a 30-day period and timeline followback at the 30-day followup, supporting validity of mobile technology for assessment of alcohol use.	Bernhardt et al. 2005, 2007, 2009
Tiplady and colleagues, University of Edinburgh, Edinburgh, United Kingdom	N/A	Text messages remind participants to complete cognitive assessments and inquire about alcohol use	Twice-daily cognitive assessments, followed by a two-period crossover lab study (*n* = 38)	None noted	Moderate drinkers	Mobile phones allowed practical research on cognitive performance in everyday setting.	Tiplady et al. 2009
Moore and colleagues, Cardiff University, Cardiff, United Kingdom	N/A	Text messages collect daily alcohol consumption and deliver feedback intervention on estimated alcohol expenditures	Feasibility study (*n* = 82) and exploratory RCT (*n* = 86); Intervention: text-message drinking survey plus drinking expenditure feedback; Control: text-message drinking survey	Cites prior empirical evidence on text-messaging monitoring studies	College students	Self-reported alcohol consumption data was significantly associated with severity scores obtained using formal screening instruments. Attrition was not associated with greater alcohol use. Text messaging was acceptable to participants and preferred over email and Web-based methods. The exploratory RCT results showed that the reduction of drinking in the intervention group warrants a future large-scale RCT study.	Moore et al. 2013
**Text-Messaging Intervention Systems**							
Agyapong and colleagues, University of Alberta, Alberta, Canada	N/A	Supportive text messaging; messages designed to improve mood and offer support for alcohol abstinence	RCT (*n* = 54); Intervention: daily support text messages Control: fortnightly thank-you text message	Cites prior empirical evidence on text-messaging interventions	Patients with alcohol use disorders and comorbid depression	High retention and perceived usefulness among intervention-group participants; significantly lower depression reported in intervention group compared with the control group; no effect on cumulative abstinence or depression score at 3-month postintervention.	Agyapong et al. 2012, 2013
Irvine and colleagues, University of Dundee, Scotland, United Kingdom	N/A	36 text messages; 9 of these messages asked questions	Feasibility study (*n* = 67)	Communication theory; social cognition model; motivational interviewing; transtheoretical model of behavior change	Socially disadvantaged men	88% of participants responded to text messages; little attenuation in followup; participants engaged with text messages and provided personal responses.	Irvine et al. 2012
Stoner and Hendershot, Talaria, Inc., Seattle, Washington	Adaptive goal-directed adherence tracking and enhancement (AGATE) system	Text messages sent based on self-reported adherence patterns	RCT (sample size not reported); Intervention: AGATE Control: structured alcohol and side effects diary	Medication-adherence literature and empirical evidence from the literature on assessment methods	Treatment-seeking heavy drinkers who take naltrexone	N/A (currently in trial phase)	Stoner and Hendershot 2012
Alessi and Petry, University of Connecticut, Storrs, Connecticut	N/A	Video recording using breath analyzer; contingency management (increased rewards for not drinking)	RCT (*n* = 30) Intervention: increased compensation if nondrinking; Control: same compensation for any drinking status	Contingency management using tangible incentives	Regular drinkers (non– alcohol dependent)	Increased percentage of patients who provided a negative drinking sample and reduced self-reported number of drinking days	Alessi and Petry 2013
Weitzel and colleagues, Emory University, Atlanta, Georgia	N/A	Tailored messages based on self-reported drinking status and consequences	RCT *(n* = 40); Intervention: daily survey via a handheld computer plus tailored messages; Control: daily survey via a handheld computer only	Cites prior empirical evidence on text messaging interventions	College students	Fewer drinks per drinking day and lower expectancies to get into trouble as a result of alcohol consumption among intervention group participants compared with those in the control group.	Weitzel et al. 2007
Mason and colleagues, Virginia Commonwealth University, Richmond, Virginia	N/A	Tailored messages based on baseline survey response	RCT (*n* = 18) Intervention: text messages; Control: no messages	Motivational interviewing	College students	Increased readiness to change drinking behavior among intervention-group participants compared with those in the control group.	Mason et al. 2014
Suffoletto and colleagues, University of Pittsburgh, Pittsburgh, Pennsylvania	N/A	Weekly text message–based feedback with goal setting (intervention)	Three-arm RCT (*n* = 45); Control: uniform message reminding of the final survey; Assessment: text message–based drinking survey; Intervention: same as assessment plus tailored text-message response	NIAAA recommendations for alcohol brief interventions, customized based on individual responses	Young adults (ages 18–25 years) presenting to the emergency department	Compared with baseline, intervention group had 3.4 fewer heavy drinking days (HDDs) and 2.1 fewer drinks per drinking day (DPDDs) in the last month, whereas the assessment group had 1.8 more HDDs and 1.1 more DPDDs and the control group had 1.1 fewer HDDs and 0.6 fewer DPDDs.	Suffoletto et al. 2014
**Comprehensive Recovery Management Systems**							
Dulin and colleagues, University of Alaska, Anchorage, Alaska	LBMI-A (Buddy System)	Assessment and feedback; highrisk locations; supportive people; craving-coping strategies; communication skills training; pleasurable activities	Pilot study (*n* = 52): Intervention: LBMI-A; Control: publicly available Web-based intervention plus bibliotherapy	Motivational enhancement; community reinforcement	Adults (ages 18–45 years) with alcohol use disorders not in other types of treatment	Both interventions resulted in significant and large decreases in HDDs and DPDDs (LBMI-A group evidenced a 60 percent drop in HDDs over 6 weeks). LBMI-A group evidenced more rapid change in first month of use and had better retention than the Web-based intervention.	Dulin et al. 2013, 2014*a*,*b*; Gonzalez and Dulin 2014
Gustafson and colleagues, University of Wisconsin, Madison, Wisconsin	A-CHESS	Weekly check-in; panic button; My Team; team feed; news; recovery information; AA/NA meeting locator; My Messages; easing distress	RCT (*n* = 349) Intervention: A-CHESS plus usual care; Control: usual care	Self-determination theory; Marlatt’s relapse model	Alcohol-dependent patients exiting residential treatment	Intervention-group patients reported reduced risky drinking days by 57 percent compared with the control group.	Chih et al. 2014; Gustafson et al. 2014
**Gaming systems**							
Gamito and colleagues, Lusophone University of Humanities and Technologies, Lisbon, Portugal	N/A	Cognitive games on mobile phone systems	RCT (*n* = 54); Intervention: games plus usual care; Control: usual care	Cognitive rehabilitation	Alcohol-dependent patients	Patients in the intervention group showed improved frontal lobe functions compared with those in the control group.	Gamito et al. 2014

## Footnotes

1 The search of electronic literature databases used a combination of two keyword sets: (1) “App” OR “Apps“ OR “mobile application*” OR “mobile health” OR “mhealth” OR “text” OR “texting” OR “text message*” OR “messaging” OR “smartphone* OR “iphone*” OR “Android” OR “mobile device*” and (2) “alcohol addiction” OR “alcohol abuse” OR “alcohol dependen*” OR “alcoholic” OR “alcoholism” OR “alcohol* recovery” OR “sobriety” OR “sober” OR “addiction recovery.” (An asterisk is used in a search term to include any string of letters that follows it; for example, searching on “alcohol dependen*” would produce search results that include both “alcohol dependent” and “alcohol dependence”.) Articles were retrieved from several databases, including EBSCO HOST (including PsychInfo and Academic Search Premier), Web of Knowledge, and Engineering Village.

2 It should be noted that the categorization is somewhat subjective and may not have categorized all systems appropriately.
